# Scalable Passive Sleep Monitoring Using Mobile Phones: Opportunities and Obstacles

**DOI:** 10.2196/jmir.6821

**Published:** 2017-04-18

**Authors:** Sohrab Saeb, Thaddeus R Cybulski, Stephen M Schueller, Konrad P Kording, David C Mohr

**Affiliations:** ^1^ Center for Behavioral Intervention Technologies Department of Preventive Medicine Northwestern University Chicago, IL United States; ^2^ Rehabilitation Institute of Chicago Department of Physical Medicine and Rehabilitation Northwestern University Chicago, IL United States

**Keywords:** sleep monitoring, mobile phones, decision trees, classification

## Abstract

**Background:**

Sleep is a critical aspect of people’s well-being and as such assessing sleep is an important indicator of a person’s health. Traditional methods of sleep assessment are either time- and resource-intensive or suffer from self-reporting biases. Recently, researchers have started to use mobile phones to passively assess sleep in individuals’ daily lives. However, this work remains in its early stages, having only examined relatively small and homogeneous populations in carefully controlled contexts. Thus, it remains an open question as to how well mobile device-based sleep monitoring generalizes to larger populations in typical use cases.

**Objective:**

The aim of this study was to assess the ability of machine learning algorithms to detect the sleep start and end times for the main sleep period in a 24-h cycle using mobile devices in a diverse sample.

**Methods:**

We collected mobile phone sensor data as well as daily self-reported sleep start and end times from 208 individuals (171 females; 37 males), diverse in age (18−66 years; mean 39.3), education, and employment status, across the United States over 6 weeks. Sensor data consisted of geographic location, motion, light, sound, and in-phone activities. No specific instructions were given to the participants regarding phone placement. We used random forest classifiers to develop both personalized and global predictors of sleep state from the phone sensor data.

**Results:**

Using all available sensor features, the average accuracy of classifying whether a 10-min segment was reported as sleep was 88.8%. This is somewhat better than using the time of day alone, which gives an average accuracy of 86.9%. The accuracy of the model considerably varied across the participants, ranging from 65.1% to 97.3%. We found that low accuracy in some participants was due to two main factors: missing sensor data and misreports. After correcting for these, the average accuracy increased to 91.8%, corresponding to an average median absolute deviation (MAD) of 38 min for sleep start time detection and 36 min for sleep end time. These numbers are close to the range reported by previous research in more controlled situations.

**Conclusions:**

We find that mobile phones provide adequate sleep monitoring in typical use cases, and that our methods generalize well to a broader population than has previously been studied. However, we also observe several types of data artifacts when collecting data in uncontrolled settings. Some of these can be resolved through corrections, but others likely impose a ceiling on the accuracy of sleep prediction for certain subjects. Future research will need to focus more on the understanding of people’s behavior in their natural settings in order to develop sleep monitoring tools that work reliably in all cases for all people.

## Introduction

### Background

Sleep is intrinsically linked to many aspects of life, including both physical and mental health [1]. The connection between sleep and well-being is bidirectional, where sleep disorders can cause significant burden on a person’s life, and underlying disease can manifest itself as disruptions a person’s sleep. These links manifest themselves in a number of facets of a person’s health, from immune and metabolic effects [2] to disordered sleep patterns as a part of disease processes such as schizophrenia, depression, or post-traumatic stress disorder [3,4]. As such, sleep can provide a unique window into monitoring, tracking, or treating disease processes, and be both a target and outcome of intervention [5]. Thus, monitoring sleep is important.

Indeed, sleep monitoring plays a critical role in current clinical practice. Polysomnography, the “gold standard” for diagnosis of sleep disorders, monitors a variety of signals over the course of several nights, for example, electroencephalogram (EEG), breathing, and muscle and eye movements, to produce a detailed picture of a patient’s sleep patterns [6]. Ambulatory polysomnography is a lower cost option than in-clinic assessment and acquiring data from a person’s home environment might better represent their typical sleep patterns [7]. However, it is still expensive, time-consuming, and the tools used for assessment might themselves interact with the sleep behavior. Thus, for chronic sleep tracking, clinicians have typically relied on instruments such as sleep diaries, questionnaires, and similar instruments [8]. These approaches have several drawbacks, such as patient adherence and reporting bias [9]. Having a way to monitor sleep that does not suffer from these drawbacks but is easier to perform than polysomnography would be a boon for clinical practice and research.

With the advent of mobile phones, a majority of Americans now carry a multifunctional sensor platform in their pocket [10]. These devices and other wearable activity sensors can be used to monitor a person’s behavior and environment, and as a result, can be used to monitor sleep. Previous work has used this mobile-sensor-based approach to predict sleep with relatively high accuracy. One study predicted the sleep or awake state of every 10-min long bin of phone sensor data with 93% accuracy [11]. Another study estimated the sleep duration with an error of 42 min [12], and a subsequent study on a college student population was able to predict bedtimes with an accuracy of 25 min of the ground truth [13]. Finally, a more recent study was able to predict sleep or awake states with 89% accuracy solely based on the users’ interactions with their phones [14]. These approaches hold promise for sleep tracking in the future; however, there remains significant work to do before they can be used more generally.

Several issues impair the ability to apply these findings to the general population. First, much of this work has used small subsets of the population, mostly students [12]. Students tend to be homogeneous in terms of demographics such as age and other patterns such as school schedules, and some evidence suggests that these demographic and life similarities might impact their sleep patterns [9,15]. Second, study participants typically receive instructions, such as placing the mobile phone face-down on the bed with them as they went to sleep [12] or keeping the phone turned on and to keep it in their bedroom while sleeping [11]. Although this increases the reliability of automated sleep assessment, to the degree that people to change their daily habits, it means that attempts to use these assessments in the wild will likely fail. Finally, many studies simply exclude noncompliant participants in the analysis. However, noncompliance may be related to other factors, such as untraditional sleep schedules, that might bias results in ways that reduce generalizability [13]. Therefore, classifiers (algorithms that distinguish sleep from waking states) that do not depend on specific instructions regarding the use or placement of the phone and which are generalizable to a broader population still need to be tested.

### Aim of This Study

In this study, we aim to explore the use of mobile devices for sleep tracking in a broad population of participants. Participants are recruited from across the United States without restrictions on age, leading to a substantially more heterogeneous sample than previous work. Participants use their own personal devices and are given no instructions on device use, allowing us to gather data from the natural, daily course of their lives. We will use techniques from machine learning to detect the sleep times of each participant, and will examine whether these techniques will generalize to other participants. Overall, we will assess if, and to what extent, we can scale passive sleep monitoring, from normal everyday phone use, to the more general population.

## Methods

### Participant Recruitment

We recruited the participants for our study between October 28, 2015 and February 12, 2016. The recruitment was done in collaboration with Focus Pointe Global (FPG), a company that specializes in market and scientific research strategies and participant recruitment and retention. FPG used Internet and qualitative panels of participants as a primary means of recruitment. They sent out emails to these panels with links to the screener questionnaire. Additionally, they used phone calls to potential participants in their in-house registries.

In the screener questionnaire, interested individuals were screened for eligibility. Individuals were eligible for our study if they were at least 18 years old, able to read and understand English, owned a mobile phone with Android 4.4 through 5.1 (excluding 5.0 due to problems that limited reliable access to some sensor data), and had access to WiFi for at least one 3-h period a day. We excluded individuals who were diagnosed with any psychotic disorders, were identified as not being able to walk more than half a mile (4 city blocks), or had positive screens for alcohol abuse (alcohol use disorder identification test, AUDIT [16] score ≥16), drug abuse (drug abuse screening test, DAST-10 [17] score ≥6), suicidal ideation (patient health questionnaire-9 item, PHQ-9 [18] item 9 rating ≥1; Beck depression inventory, BDI-II [19] item 9 rating ≥2), or bipolar disorder (mood disorder questionnaire, MDQ [20] question 1 score ≥7, an endorsement of question 2, and a response of 2 or 3 for question 3). We also excluded those individuals who shared their phone with others. Eligible participants were consented using procedures approved by the Northwestern University Institutional Review Board, which included descriptions of the data to be gathered along with data security and privacy policies. We selected roughly equal numbers of participants in four groups, such that there were wide ranges of depression and anxiety symptoms in the sample. We defined the groups as depressed and anxious (PHQ-9 ≥10; generalized anxiety disorder-7 item, GAD-7 ≥10), depressed only (PHQ-9 ≥10; GAD-7 <10), anxious only (PHQ-9 <10; GAD-7 ≥10), and healthy (PHQ-9 <10; GAD-7 <10).

Each participant was enrolled for a period of 6 weeks. First, a study ID was assigned to the participant by FPG. Then participants were asked to complete a Web-based questionnaire consisting of demographics (eg, age, gender, race and ethnicity, state of residence) and life aspects (eg, living situation, employment issues, where they keep their phone) that could potentially affect their sleep and phone use. Participants were compensated from US $25 to US $270.40 depending on how long they stayed in the study, and how much of the daily questionnaires they answered.

### Data Collection

We collected two categories of data: mobile phone sensor data and ecological momentary assessment (EMA) data, which consisted of daily questions sent to participants asking them about their last night sleep times. The sensors used in our study and their attributes are listed in Table 1.

**Table 1:** List of the mobile phone sensors and their attributes, used in our study.

Sensor	Description
Activity	Physical activity class provided by the Android Activity Recognition API^a^ (*still, walking, running, tilting, on bike, in vehicle, unknown*) and the confidence of the classifier (0-100%)
Light	Light intensity (lux)
Sound	Average sound intensity (dB) and dominant sound frequency (Hz)
Screen	State of the phone screen (on or off)
Battery	State of the battery (*not charging*, *charging via power cable*, *charging via USB*^b^)
GPS^c^ location	Geographic latitude and longitude in degrees
WiFi	The MAC^d^ address of the access point which the device is currently connected to
Communication events	Contact names, contact numbers, outgoing or incoming calls, outgoing or incoming SMS^e^
Time of day	Time of the day

^a^API: application program interface.

^b^USB: Universal Serial Bus.

^c^GPS: Global Positioning System.

^d^MAC: media access control.

^e^SMS: short message service.

EMA data was collected on a daily basis. On each day, at 9am local time, the questionnaire was launched on each participant’s phone, asking them about the time they went to sleep last night, or *sleep start time*, and the time they woke up, or *sleep end time*. Participant could respond immediately to the questions, or delay the response until later that day. If they did not answer the questions before 12am that night, the questionnaire disappeared; and on the next day, a new questionnaire was launched asking about sleep start and end times of the night before. *Sleep duration* was defined as the time from sleep start time to sleep end time.

We used Purple Robot [21] to collect both sensor and EMA data. Purple Robot is a multipurpose, open-source Android app that is developed for our phone-based behavioral sensing studies [22], and adapted to this study. The app gathers data from the sensors available on the phone, initially stores them locally on the device, and then transmits them as network connectivity becomes available. This allows data collection in a variety of wireless connectivity scenarios with the confidence that intermittent network access does not affect the nature, quality, or quantity of the collected data.

Purple Robot anonymized sensitive information before storage and transmission. Specifically, it used a standard MD5 hashing algorithm [23] to anonymize the contact names and numbers in the communication events sensor (see Table 1), as well as the participant IDs. Once the data was anonymized, it was locally stored on the device, transmitted to secure data collection server via encrypted, password-protected tunnels, and then deleted from the device. The mobile phone data residing on the server could be linked with other information gathered during the study only if the unique identifiers used by the participants and the study-specific keys used to encrypt the data were known. Furthermore, these were only accessible to individuals with the proper credentials. Overall, these security measures helped to protect the participants’ privacy, particularly regarding sensor data such as GPS and MAC addresses, which could risk leakage of personal information.

Initial tests showed that the sound sensor (microphone) was draining battery power to a considerable degree, which could interfere with our data collection and dissatisfy the participants. Thus, we sample the microphone every 5 min for 30 s at a time. The Purple Robot sound sensor then reported the average sound amplitude (dB) and the dominant sound frequency during that 30 s period. The dominant frequency was calculated by taking the Fast Fourier Transform (FFT) of the signal, and finding the frequency at which it was maximum. Using this procedure, we considerably decreased the battery power consumption by Purple Robot.

### Feature Extraction

Before using the collected phone sensor data for developing sleep detection algorithms, we extracted their attributes, or *features*. To extract features, we first divided all sensor data into 10-min-long windows. Then, from each window, we extracted 22 distinct features as listed in Table 2. The choice of 10 min was made for consistency with previous research [11]. In our feature set (Table 2), we included features that had previously been shown to be useful [11-13]. For the location features, *location variance,* and *location change*, we converted the GPS coordinates in latitude and longitude degrees to 2D coordinates in kilometers, using the method described in [24], before extracting the features. In addition, we also included time of the day as a feature, since we hypothesized that the time alone is a strong predictor of whether a person is asleep or awake.

To deal with missing sensor data, we used different strategies for different sensors. For the communication events and the screen sensors, we used a 0 value when data was not present, as for these cases absence of data meant no events. For the activity sensor, since the Android’s Activity Recognition API (application program interface) does not generate new samples when the phone has been in the same state for a long time, we filled the missing points with the activity sample from the last window which contained data. For the rest of sensors, if the window was empty, the corresponding features were set to “Not a Number” (*NaN*).

**Table 2:** List of features used in the study.

Feature	Description
Stillness	Percentage of *still* activity
Light power	Mean of light intensity
Light range	Range of light intensity
Light kurtosis	Kurtosis of light intensity
Light change	< (*L* (*t*) – *L* (*t*-1))^2^ / *L* (*t*-1)^2^ > *L(t)*: light intensity at time *t*, and <.> denotes the average over time.
Audio power	Mean of audio power
Audio freq min	Min. dominant audio frequency
Audio freq max	Max. dominant audio frequency
Screen activity	Number of screen ON or OFF events excluding the ones that last less than 30 s
Location variance	√(*σ*^2^_lat_ + *σ*^2^_lng_) where *lat* and *lng* are latitude and longitude values in kilometers, respectively.
Location change	Average of change (as defined for light change) between latitude and longitude
Battery charging	1 if mode of battery state is *charging*; 0 otherwise
Battery USB^a^	1 if the phone is connected to USB, 0 otherwise
Battery level	Average battery level (0-100)
WiFi	Mode of WiFi MAC^b^ address (converted to integer by summing up the characters)
Last name	Last contact name (encrypted) contacted by either call or SMS^c^
Last number	Last phone number (encrypted) contacted by either call or SMS
Call	Number of phone calls
SMS	Number of SMS
Outgoing call	Number of outgoing phone calls
Outgoing SMS	Number of outgoing SMS
Time of day	Time of the day in hours (0-24), defined as the midpoint in the window

^a^USB: Universal Serial Bus.

^b^MAC: media access control.

^c^SMS: short message service.

### Sleep Detection

#### Overview

We trained algorithms to detect the sleep start and wake-up times of each participant from the sensor features extracted from their phones. These algorithms, also called *classifiers*, determined whether each feature sample, extracted from 10 min of sensor data, was from a sleep or an awake state as reported by participants. After training, the classifiers were able to predict the state of a given feature sample.

The sleep detection procedure had two stages: first, we used *random forests* to estimate the probability for a feature sample to be from sleep or awake states. Then, a hidden Markov model (HMM) used the sequence of these probabilities to determine whether the participant’s state was actually sleep or awake. In the following, we will give a more detailed description of these two stages.

#### Estimating State Probabilities

To estimate the probability for each feature sample being from an awake or asleep state, we used ensembles of decision trees known as random forests [25]. Each tree in a random forest makes a prediction, or vote, about the class of the feature sample. The random forest calculates the class probability by averaging over the predictions of individual trees. In this study, we used 50 trees.

We trained the random forest to estimate the state (*awake* or *sleep*) probabilities based on the last 5 feature sets extracted from the last 5 windows. For training, we used the bagging method [26], which randomly samples the dataset with replacement to create a training set for each tree. In this way, each tree only observes part of the dataset. In addition, each decision node in the tree randomly samples 5 out of 22 features, and finds the best feature and the best split value based on a Fisher information gain criterion. Therefore, each tree only observes part of both the data samples and the features. This makes random forests less prone to overfitting and a better candidate for generalization to unseen data [27].

#### Determining States

Although our random forest classifiers use the last 5 feature samples to provide the class probability of the current sample, they ignore the class probabilities of the surrounding samples. This disregards the fact that sleep and awake states change slowly over time. In fact, transitions from *awake* to *sleep* and vice versa usually happen once in a given 24-h period. Therefore, it is important to consider the class probabilities of the neighbor samples in calculating the class probability of any given sample.

To determine the *sleep* or *awake* states, we first use a median filter in order to reduce the effect of fast changes in the class probabilities. A median filter replaces each sample by the median of *w* neighboring samples. Here, we set *w*=21, corresponding to 210 min in the data. After recalculating the probabilities, we use the threshold of 0.5 to determine the class of each sample (Probability≤.5: *awake*; Probability>.5: *sleep*). In this way, the median filter captures the slower dynamics of the state probabilities.

After recalculating the state probabilities, the next step is to determine the states. For this, we use a HMM, which is a Bayesian statistical model that infers the states of an unobserved variable, sleep state in our study, given a set of observations, here the set of states estimated by the median filter. The HMM uses a set of parameters called transition probabilities, which represent the probability of transition between the classes. Because there are typically only one sleep-to-awake and one awake-to-sleep transition in each 24-h period, and given that we have 144 feature samples in each 24-h period, we set the transition probabilities as the following:

*T*_(sleep-awake)_= *T*_(awake-sleep)_=1/144

*T*_(sleep-sleep)_= *T*_(awake-awake)_=143/144

#### Training and Cross-Validation

We train sleep detection models in two different ways: (1) global models and (2) personal models. The former is trained on all data from a number of participants and cross-validated on the rest, whereas the latter is trained and cross-validated on the data from the same participant at different times.

For the global models, we use a subject-wise, 10-fold cross-validation method. We first divide the participants into 10 almost equal, nonoverlapping sets. Then, we train models on all sets except one and cross-validate it on the remaining set. We repeat this procedure 10 times so that all participants are used for cross-validation.

To train personal models, we divide each participant’s data into 3 nonoverlapping folds. Then, we train models on 2 folds and validate them on the remaining fold. We repeat this procedure 3 times until all folds have been used for validation. The classification accuracy was averaged across the folds, representing the classification accuracy for the subject.

## Results

### Participants

**Figure 1:**
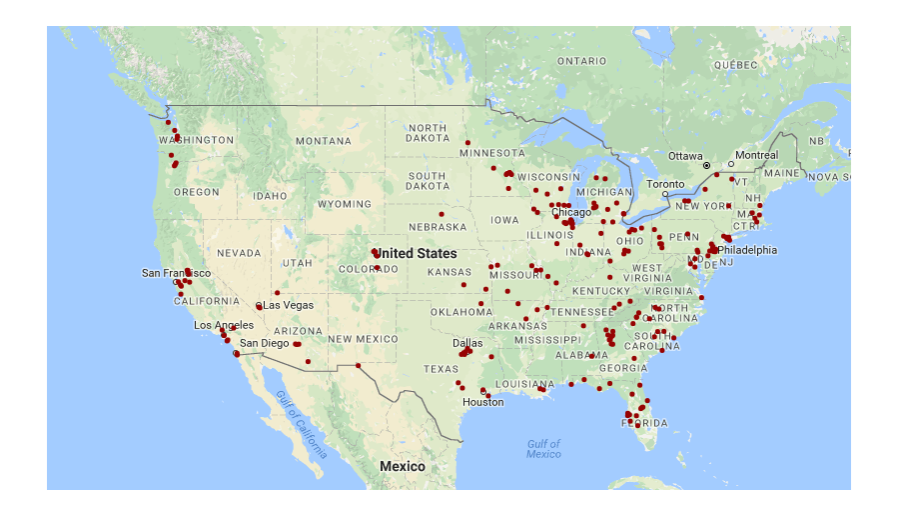
Locations of participants on the map, shown as red dots. We added a small random value, drawn from a Gaussian distribution with zero mean and standard deviation of 1.5 km in geographic distance, to each participant’s location so that their exact coordinates cannot be extracted from the figure.

In total, 208 eligible participants were recruited for the study. One participant did not install the software on their phone, and therefore was removed from the analysis. Of the 207 participants included in the analysis, 82.6% (171/207) were females and 17.4% (36/207) were males. Their ages ranged between 18 and 66 years old, with a mean of 39.3 (SD 10.3). They represented a geographically diverse sampling of the United States, as shown in Figure 1. Participants did not perfectly represent the racial and ethnic diversity of the United States with 78.7% (163/207) Caucasian, 11.6% (24/207) African American, 2.4% (5/207) Asian, 1.4% (3/207) Native American, and the remaining 4.3% (9/207) of participants were a combination of two or more races. It was found that 1.4% (3/207) of participants preferred not to specify race and 9.2% (19/207) of participants noted Hispanic as their ethnicity. Nevertheless, this is a diverse pool of demographics and locations.

The outcomes on the questionnaires asked during the screening were as follows: the average drug abuse score (DAST-10) was 0.56 (SD 1.06), alcohol abuse score (AUDIT) was 3.66 (SD 3.35), depression score (PHQ-9) was 9.72 (SD 5.10), and anxiety score (GAD-7) was 9.01 (SD 5.41). As expected, the drug and abuse scores were low, since we excluded individuals with high scores. However, there was a wide distribution in depression and anxiety scores as it was intended in the recruitment procedure.

Participants had diverse educational backgrounds: 1.9% (4/207) of participants had some high school education, 12.1% (25/207) had completed high school, 35.3% (73/207) had some college training, 13.5% (28/207) had 2-year college training, 23.6% (49/207) had Bachelor’s degree, 11.1% (23/207) had Master’s degree, and 2.4% (5/207) had professional Doctorate degree.

Finally, we asked the participants questions about the aspects of their lives that would potentially influence sleep detection. Of the 207 participants, 14.5% (30/207) lived alone, whereas 85.0% (176/207) lived with other people, and 0.5% (1/207) did not specify. In response to the employment status question, 61.4% (127/207) were employed, 20.8% (43/207) were unemployed, 8.2% (17/207) had disability which prevented them from working, 1.9% (4/207) were retired, and 7.7% (16/207) did not specify their employment status. Of the 127 employed participants, 78.0% (99/127) had one job, 18.1% (23/127) had two, 3.1% (4/127) had three, and 0.8% (1/127) had four jobs. It was found that 87.4% (181/207) of participants mentioned that they keep their phones in their bedrooms while sleeping, whereas 12.6% (26/207) keep it in another room. It was also found that 58.5% (121/207) of participants said that they share their bedrooms with someone, whereas 41.5% (86/207) sleep alone in their bedroom. As should be expected, a broad range of life situations occurred.

In addition to understanding the lives of our participants, the purpose of collecting these data was to assist sleep detection algorithms, by adding them to sensor features as inputs. However, our initial tests showed that they were not helpful in detecting sleep, and therefore we did not use them in later analyses.

### Data Characteristics

On initial analysis of the data, there were two apparent issues that needed to be resolved. First, some participants had changed their devices during the study, with a number of them reporting on multiple devices at the same time. We detected the change in a participant’s mobile phone by tracking their device’s MAC address. Out of the 207 participants, 21 changed their phones during the study. When a participant used multiple devices at the same time, we used the data from the first device until there was no EMA data coming from that device, and then switched to the second device.

There were also inconsistent values in sensor and EMA data that needed to be corrected or removed. First, timestamps were stored in different units for some participants, due to difference in phone models. We converted the units of these timestamps to seconds which was used for all other participants. There were also out-of-range values for sleep times. For example, in some cases, we had negative sleep start or end times; these artifacts were observed in 14 of 207 subjects, with between 1 and 5 erroneous reports for each of these subjects. We removed these instances from the dataset before the analysis. After this processing, our dataset consisted of 207 subjects and a total of 10,649 reports, allowing for a broad characterization of sleep detection.

**Figure 2:**
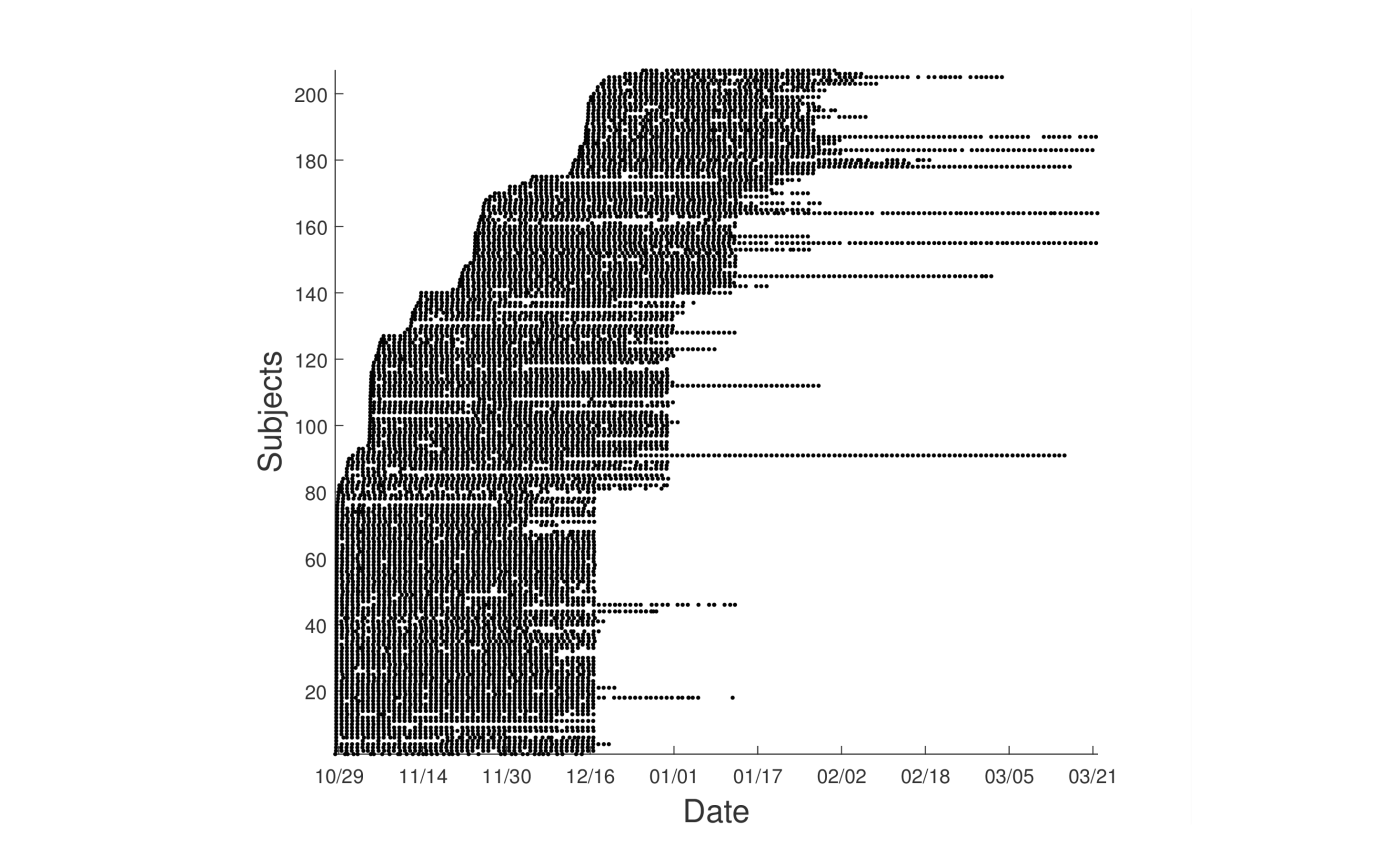
Participants' enrollment in the study, sorted based on the enrollment time. Each dot represents an ecological momentary assessment (EMA) report sample we received from the participant. The 4 recruitment waves are evident in the 4 clusters of starting times. Vertical white stripes reflect the time of day when people were less likely to complete their EMA reports (eg, night time). The number of days ranged from 11 to 137 days, with an average of 52.9 days for each participant.

For the EMA data, there was an extremely high rate of adherence, resulting in little missing data. Of the 207 participants, 10.6% (22) stopped providing labels before the end of the 6-week period. However, many continued to send data after the end of 6 weeks, with 13.0% (27/207) providing more than 60 days of data. The participants’ enrollment in the study is depicted in Figure 2. It was surprisingly doable to recruit this large number of subjects over the extended period of time of our study.

### Sleep Detection Results

**Figure 3:**
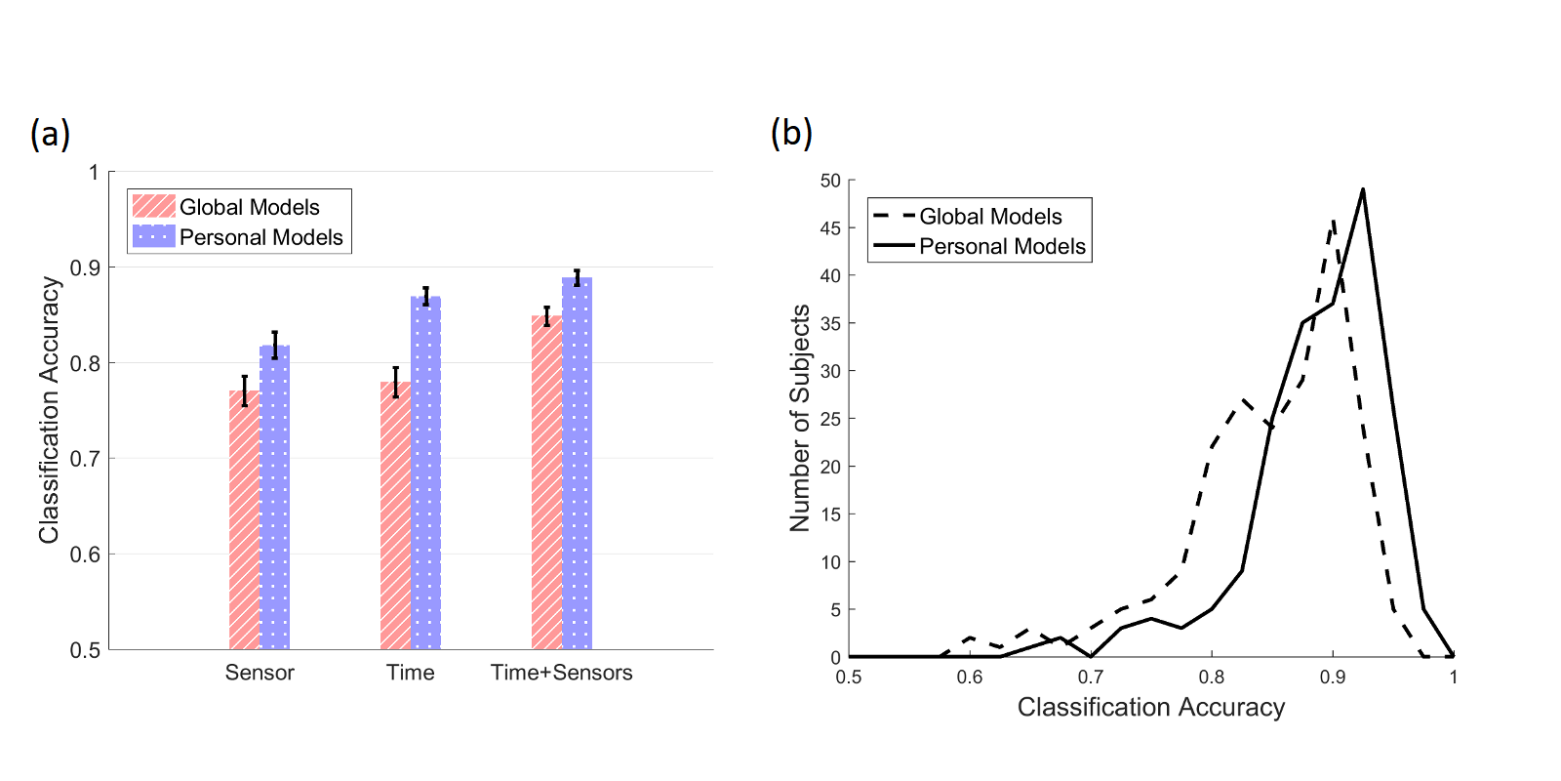
Sleep detection results. (a) Prediction accuracy (error) for global and personal models trained on time feature only, sensor-based features, and all features (see Table 2). Bars show the mean, and error bars show 95% CI. (b) Distribution of the accuracy of global and personal models trained on all features across the participants .

The average prediction accuracy of the model trained only on sensor features is about 81.8% (95%CI 81.12-82.48), and the addition of time of the day to the feature set increases this accuracy to 88.8% (95%CI 88.41-89.19; Figure 3). This accuracy, however, is only slightly better than that of the model that has only been trained on time (86.9%; 95%CI 86.68-87.12). These accuracies vary considerably across the subjects, ranging from 65.1% to 97.3% (Figure 3). Importantly, these results are in line with those of well-controlled studies for some subjects and dramatically worse for others.

**Figure 4:**
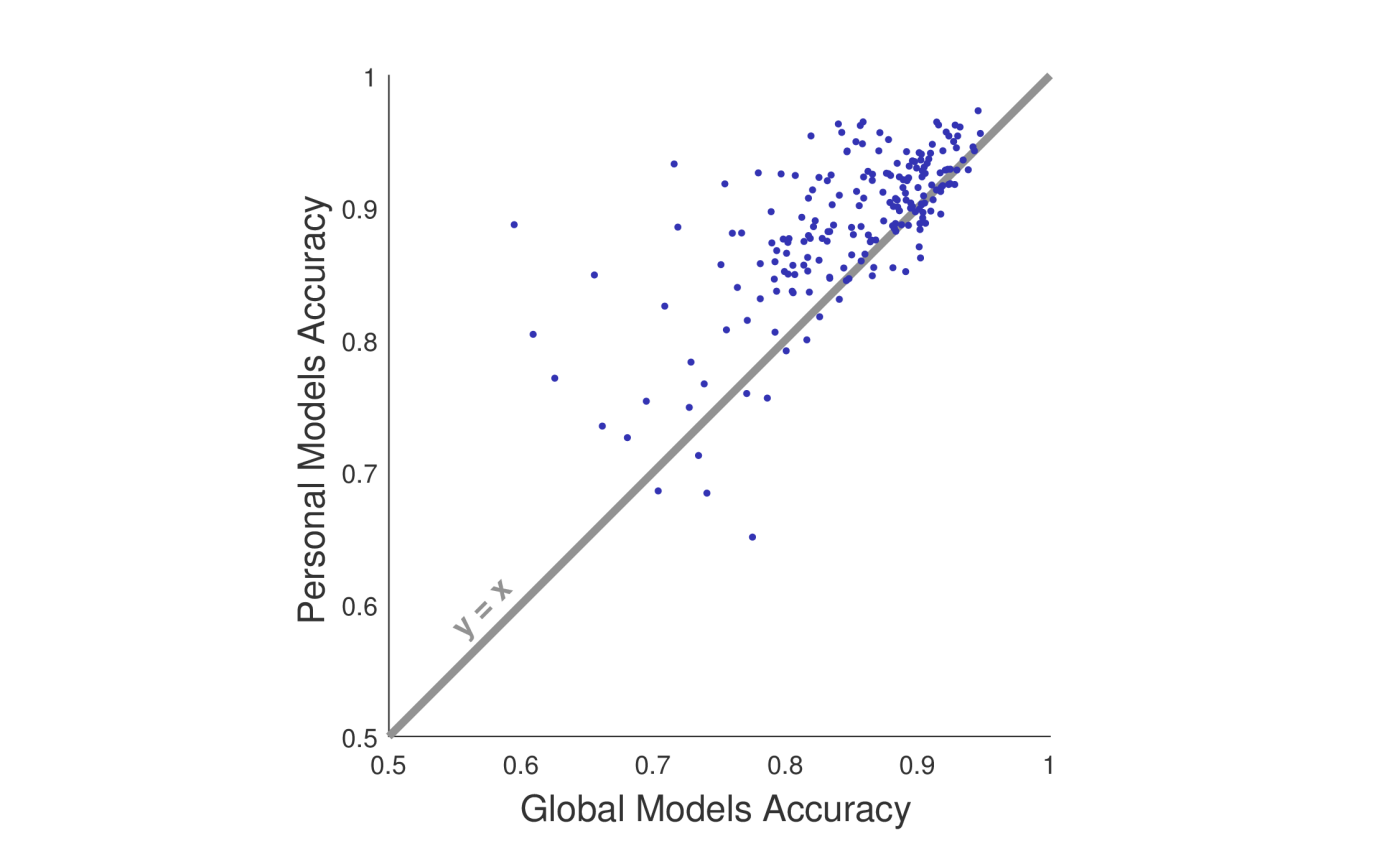
The accuracy of global and personal models across the participants. Each dot is one participant. The dots above the gray line (y=x) indicate participants for which personal model performed better than global model, and the dots below indicate the other way around. The correlation between the personal and global model accuracies is high (r=.685; P<.001).

We also compared personal models (those trained with the same participant is predicting) with global models (those trained with other participants’ data and predicting a single participant). Figure 4 displays the correlation between the accuracy of personal and global models. Personal models fared better for 80.2% (166/207) of participants; however, the difference between personal and global models was relatively small.

### Where Do Classifiers Fail?

The large variability of prediction accuracies across the participants led us to further explore why prediction fails for specific participants. Here, we looked into various metrics of data quality and investigated their relationship to the classification accuracy. The aim was to find out whether there are specific data quality issues that caused classifiers to fail, and whether we are able to improve the classification accuracy by resolving those problems.

We found two major data quality issues: missing data and misreports. In the following, we investigate each of these issues.

#### Missing Data

**Figure 5:**
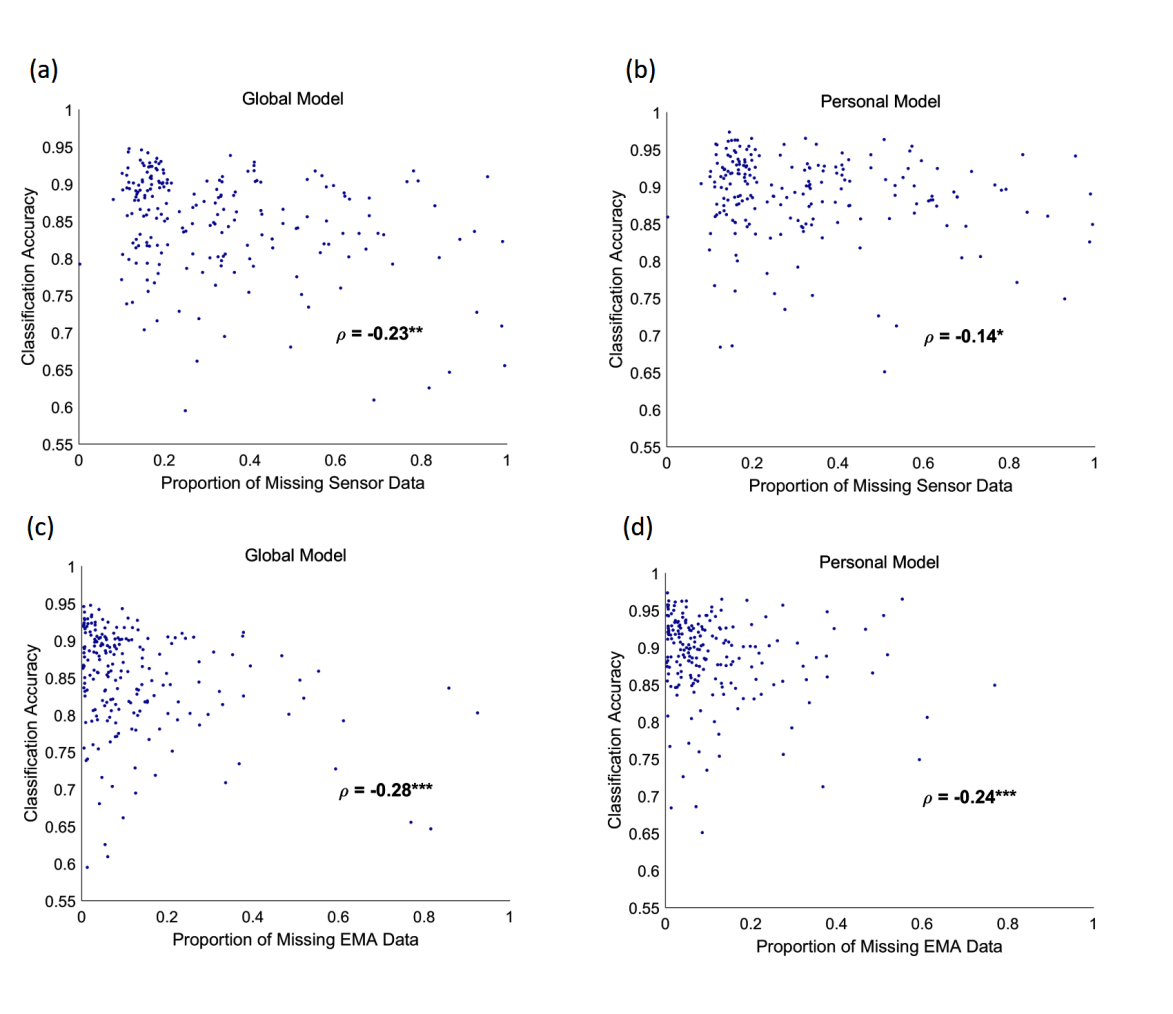
Dependence of classification accuracy on missing data. (a-b) Accuracy versus the proportion of missing sensor data for global (a) and personal (b) models. Here, we excluded the activity, communication events, and screen state sensors as their absence did not indicate missing data. (c-d) Accuracy versus the proportion of missing ecological momentary assessment (EMA) data for global (c) and personal (d) models. In all four cases, there is a weak but significant, inverse relationship between the classification accuracy and the proportion of missing data. ρ is Spearman rank correlation coefficients, with negative values indicating inverse relationships. One star indicates significance at P<.05, two at P<.01, and three at P<.001.

We estimated the proportion of missing data points in both sensor and EMA data for all participants, and we evaluated the relationship between these and classification accuracy. Although this relationship is complex Figure 5, we found that generally participants with larger proportions of missing sensor or EMA data had lower classification accuracies. Therefore, it seems that missing data is one primary cause for the classifiers’ failure.

**Figure 6:**
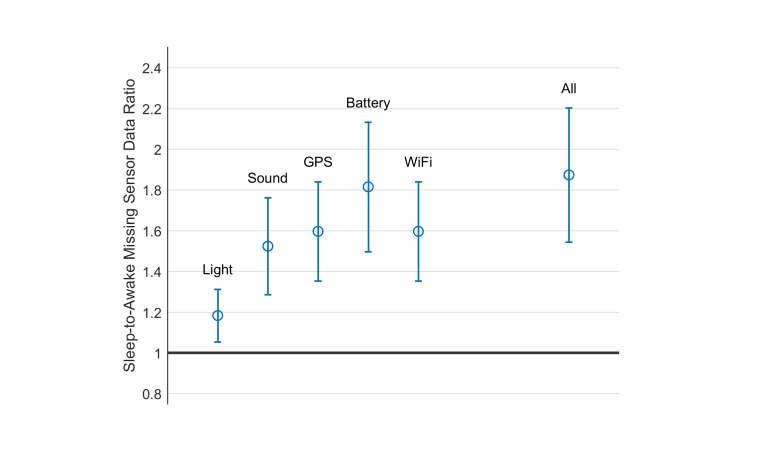
Proportion of missing sensor data during sleep states divided by the proportion of missing sensor data during awake states, across all participants.

If missing data is the major cause, the next question is when the missing samples in sensor data occurred. We estimated the proportion of sleep-state samples that were missing, as well as the awake-state samples, and calculated their ratio. As Figure 6 shows, this ratio is significantly higher than 1 for each individual sensor as well as all sensors together. For all sensors, the proportion of missing samples during sleep is almost twice compared with awake.

#### Misreports

**Figure 7:**
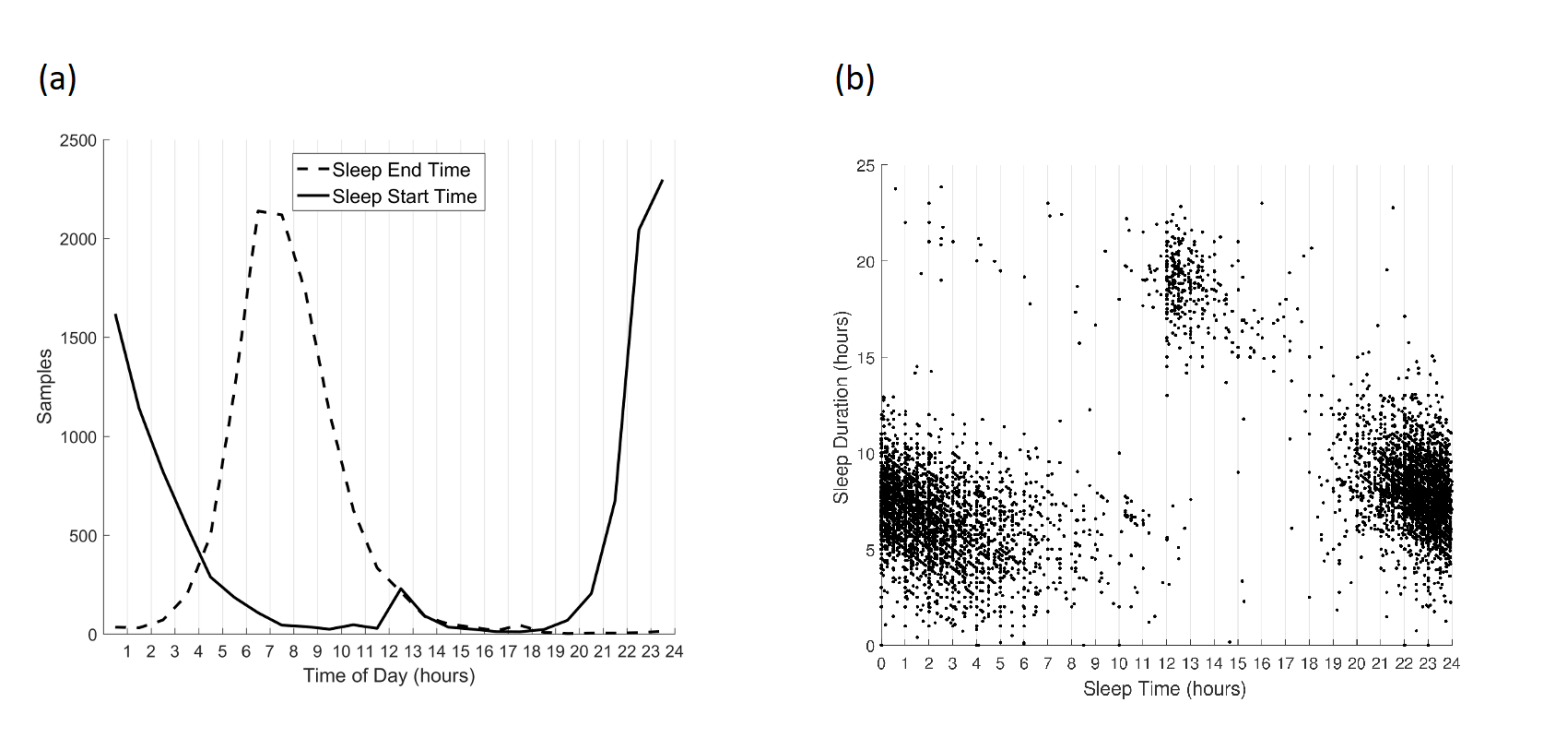
(a) The distribution of sleep start and end times. For sleep start times, there is an anomaly between 12pm and 3pm, which are likely due to the participants confusing “am” and “pm” times. (b) Sleep start times versus sleep duration, shows a distinct cluster (middle top) associated with sleep start times between 12pm and 3pm and abnormally long sleep durations (>15 h).

To investigate the possibility of misreports, we looked at the distribution of sleep start and end times. Although the distribution of sleep end times looks almost normal, sleep start times seem to have an anomaly between 12pm and 3pm (Figure 7). One possible scenario was that participants mislabeled “am” and “pm” times, especially at midnight (12am). Alternatively, these could be short mid-day naps reported instead of previous night’s sleep. To investigate which scenario was more likely, we also plotted sleep start times versus sleep duration (Figure 7). As evident in this plot, there is a distinct cluster of sleep start times between 12pm and 3pm which is associated with abnormally long (>15 h) sleep duration. Therefore, these data points could not represent mid-day naps, but they are more likely to have been caused by a confusion between “am” and “pm” in reporting sleep start times.

A summary of the data quality issues and their likely causes is shown in Table 3.

**Table 3:** Summary of the causes for low data quality which likely made the classifiers fail.

Data source	Issue	Possible causes
Sensors	Missing samples	Mobile phone off, low battery level
Sensors	Missing samples	Purple Robot, operating system, or hardware failure
Sensors	Out of range values	Device model and operating system differences
EMA^a^ reports	Missing samples	Participants not reporting
EMA reports	Abnormal values	Participants misreport

^a^EMA: ecological momentary assessment.

In addition to missing data and misreports, we also investigated whether the classification accuracy was different between participants with symptoms of depression or anxiety and the ones with no symptoms. We compared four groups of participants: nondepressed and nonanxious, depressed and nonanxious, nondepressed and anxious, and depressed and anxious. We did not find any significant difference in classification accuracy, for both global and personal models, between any of these groups.

### Improving the Quality of Data

After investigating a number of data quality issues that were likely causing the classifiers to fail in certain situations, we attempted to fix these issues and observed the effects on classification performance. Specifically, we took two steps:

1. When the reported sleep start times were between 12pm and 3pm and their associated sleep duration was longer than 15 h, we changed “pm” to “am.”
2. We removed participants for whom, on average, more than 50% of sensor samples were missing. This consisted of 20.8% (43/207) of participants.

To estimate the proportion of missing sensor data, we excluded the communication events and the screen state sensors, as their absence did not necessarily imply missing samples. After each of these steps, we trained and cross-validation both global and personal sleep prediction models.

**Figure 8:**
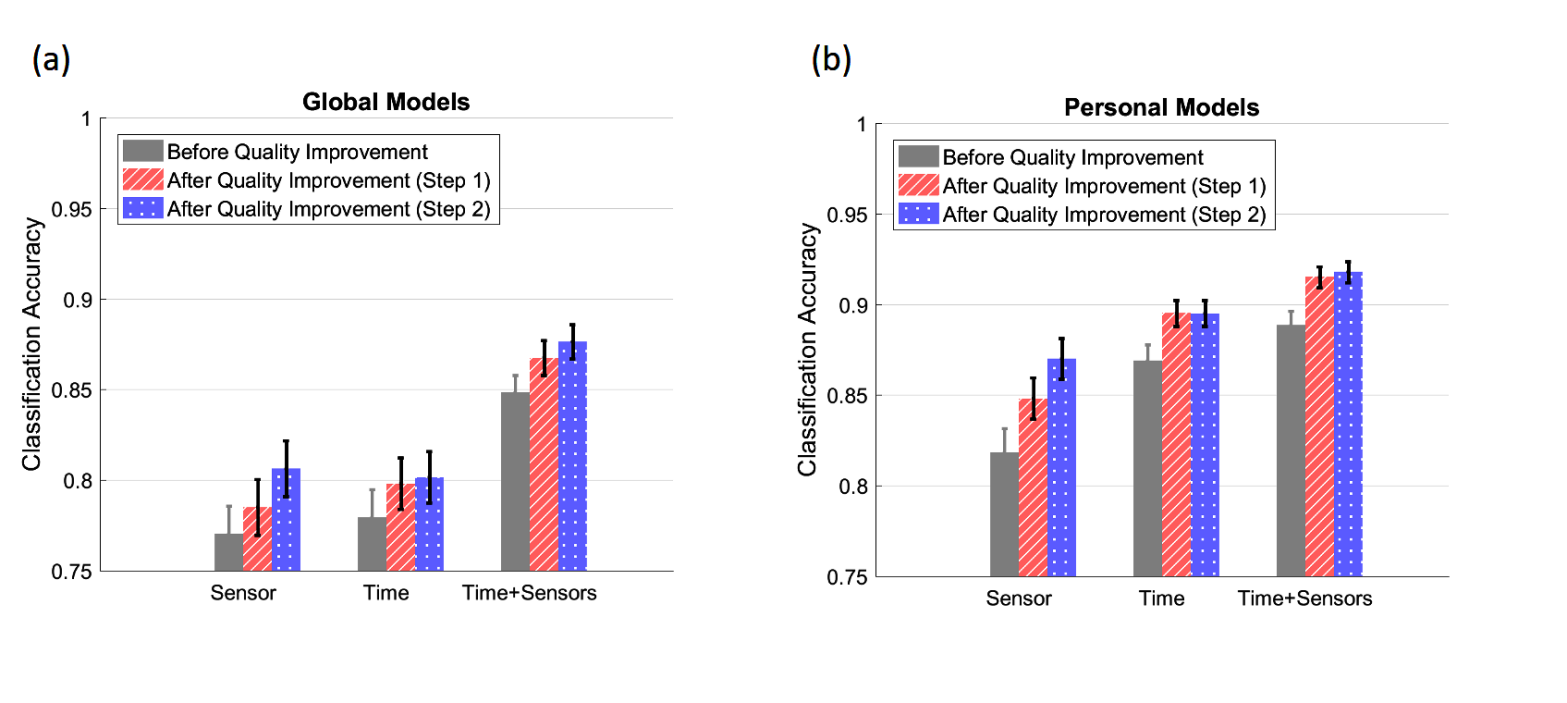
(a-b) Sleep detection results after quality improvement, for global (a) and personal (b) models. First, we corrected the reported sleep start and end times, which resulted in significantly higher accuracies (red) for all models. Then, we removed participants for whom the sensor data was missing for more than 50% of the time. This consisted of 43 participants. The resulting accuracies (blue) significantly improved for the sensor-only model, but did not change for the rest.

The results of the classifier’s performance after improving the data quality is shown in Figure 8. As the figure shows, correcting reported times considerably increased the accuracies of both global and personal classifiers, to 86.7% and 91.5%. Removing participants with large amounts of missing data further increased these accuracies, to 87.6% and 91.8%, respectively, albeit to a slightly smaller degree. It is also interesting to note that the global time-only and sensor-only models have similar performances, which is considerably lower than the performance of the global all-feature model. However, for the personal models, the accuracy of the time-only model is only slightly less than the accuracy of the model trained on both sensor features and time.

Since the amount of missing sensor data was inversely correlated with the classification accuracy, we speculated that adding an extra feature encoding the amount of missing sensor data could be beneficial. However, including these additional features did not improve the accuracy of the classifiers.

### Prediction of Sleep Start and End Times

**Figure 9:**
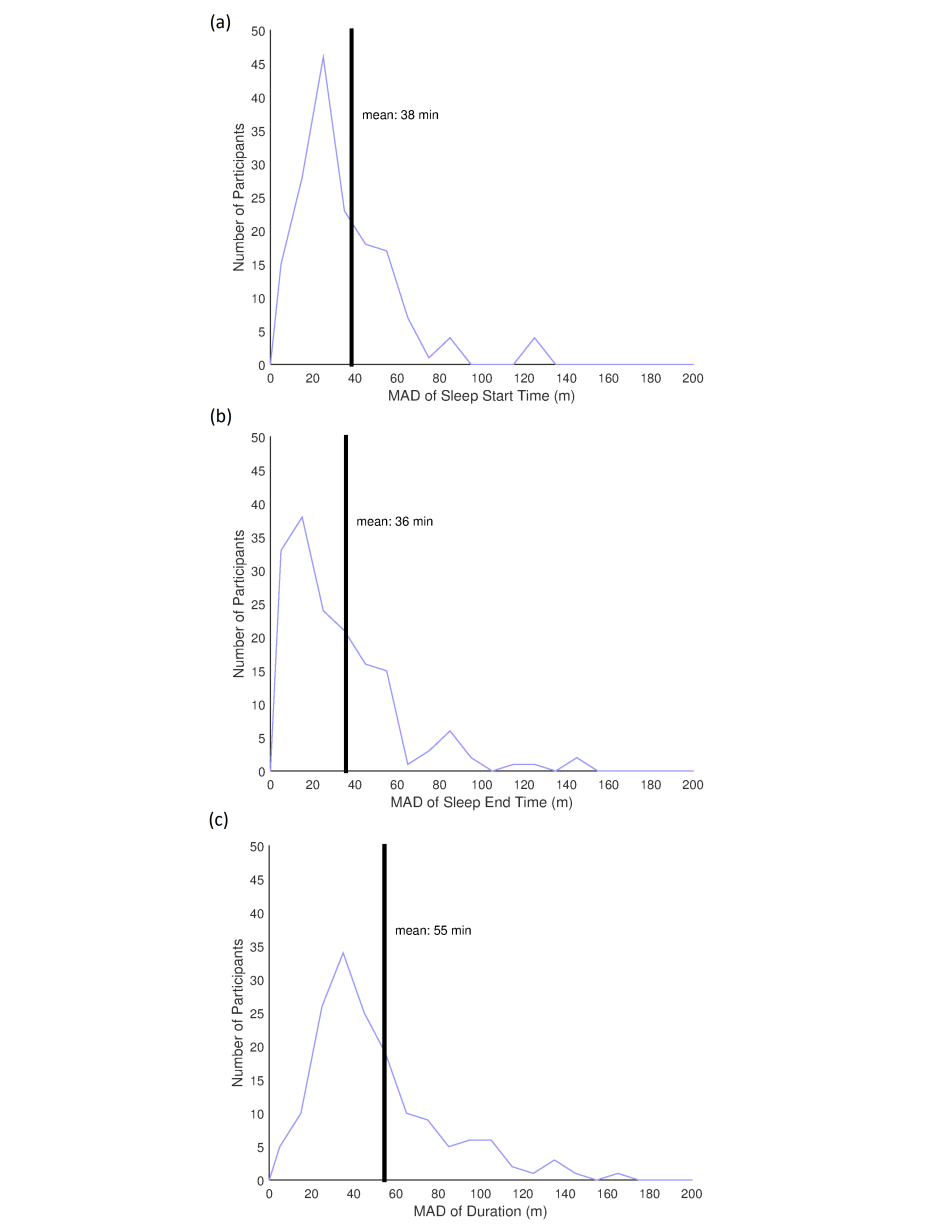
(a) Distribution of median absolute deviation (MAD) for predicted sleep start times from true sleep start times over all participants with less than 50% missing data. (b) Distribution of MAD of predicted sleep end times from true sleep end times over all participants with less than 50% missing data. Black line indicates the average MAD over those participants. (c) Distribution of MAD of predicted sleep duration from true sleep duration over all participants with less than 50% missing data. Black lines in (a)-(c) indicate the average MAD over all participants.

Using our predictions of sleep state, we can calculate values for sleep start and end times as well as sleep duration, which can be useful for monitoring clinical processes [5]. We find the closest predicted sleep period to each reported sleep period (from personal models), and examine the bin-indexed errors in predicting the start and end of that sleep period, as well as the total duration of the sleep period. These errors are all calculated on binned data, thus our minimum resolution is the bin size (10 min). We are able to estimate both sleep start and end times with approximately equal accuracy, with an average median absolute deviation (MAD) across participants of 43 min and 38 min, respectively (Figure 9). We are also able to predict sleep duration with similar accuracy, with an average MAD across participants of 58 min (Figure 9). The distribution of these errors are all relatively skew-right, which suggests that poor prediction of a small number of participants substantially affects performance.

**Figure 10:**
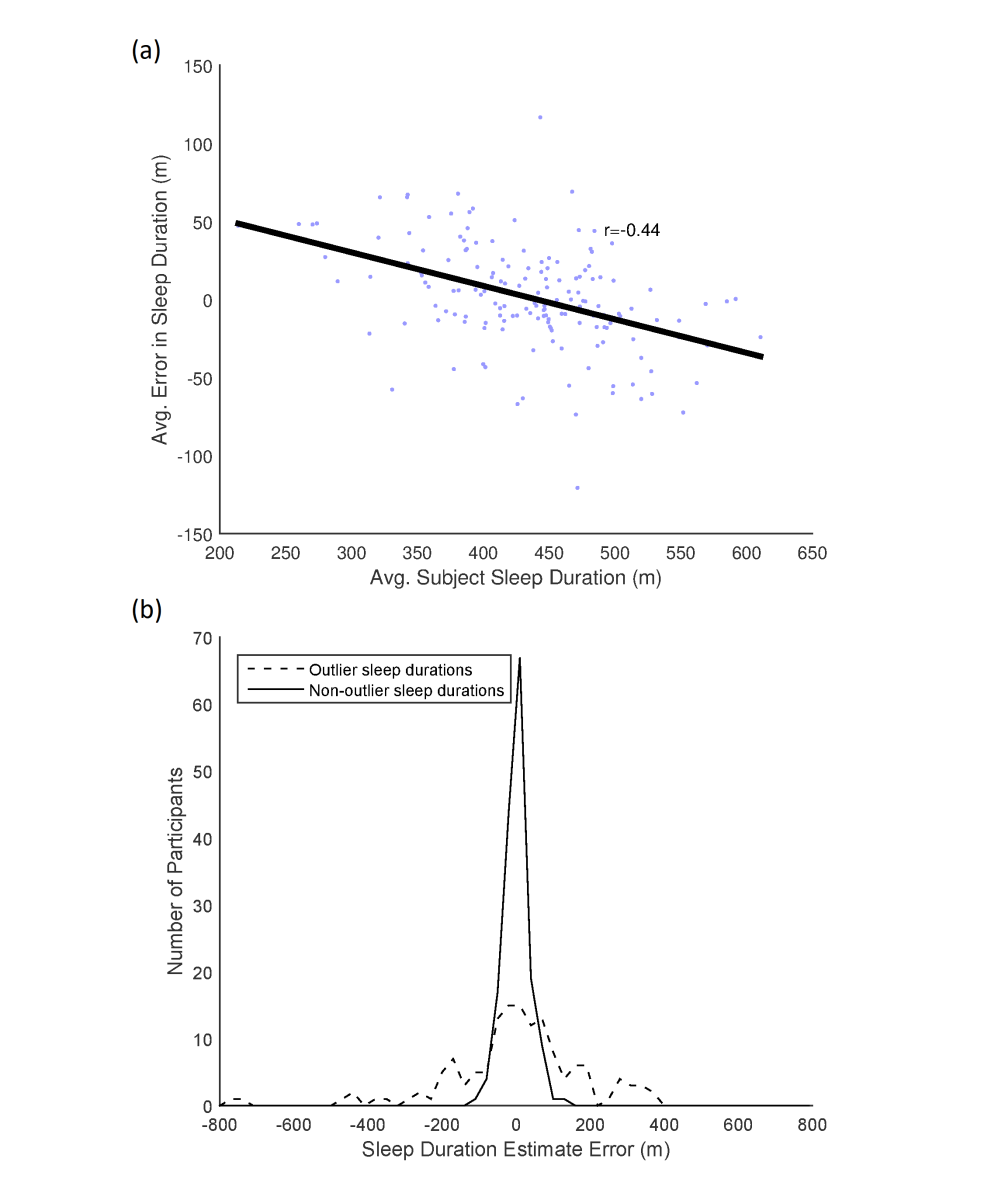
(a) Relationship between average sleep duration and average errors in estimates of sleep duration. Points reflect individual participants with less than 50% missing data, black line represents least-squares regression. (b) Distribution of average sleep duration estimation error over participants with less than 50% missing data for “outlier” (blue) and “nonoutlier” (red) sleep durations. Outlier sleep periods are defined as periods that are two standard deviations shorter or longer than the participant’s average sleep duration, and nonoutlier periods fall within those bounds.

Looking at these errors in terms of sleep characteristics can help further elucidate where we make errors. We find that participants with more extreme, that is, longer or shorter, average sleep durations have larger errors in estimating sleep duration (Figure 10). Specifically, we tend to over-estimate the duration of short sleep periods, and underestimate the duration of long sleep periods. That this occurs even with individual models suggests that, rather than a regression to a global mean, there may be something intrinsically difficult in estimating the durations of extreme sleep periods (or the sleep of those that report extreme sleep periods). We examine the per-participant performance for “outlier” (duration greater or less than two standard deviations (SDs) from the participant’s average sleep duration) and “typical” sleep periods (Figure 10). We find that, for 89% of participants, we can estimate the duration of typical sleep periods within an hour. Interestingly, we can do the same for 38.2% of participants even on their outlier sleep periods, and can estimate outlier sleep periods within 2 h for 62% of participants, suggesting that, while outlier periods are more difficult to predict regularly than most, we do not perform poorly on all outliers as a rule. This suggests difficulties in estimating the sleep duration for particular participants, which may speak to the unique challenges in estimating behavior in large, heterogeneous populations.

## Discussion

### Principal Findings

This study was a first step toward bridging initial proof-of-principle studies showing the feasibility of mobile phone-based sleep detection technology with implementation for a general population in their natural daily-life settings. We divided phone sensor data into 10-min-long windows, and calculated a number of features from them. Then, we trained our models, composed of random forests and HMMs, to predict the state of each window (*sleep* or *awake*). Although the global classifiers trained on all features were able to predict sleep state with 87.6% accuracy, personal models which were trained separately on each participant had a (significantly) higher accuracy of 91.8%. These numbers were close to the range reported by previous research in more controlled settings [11-13]. Thus our study confirms that sleep tracking via mobile devices is a viable paradigm, and that it can generalize to broad populations when used in daily-life settings.

It is interesting that the performance of personal models trained solely on the time of the day was only slightly lower than the ones trained on all features. This suggests that an individual’s sleep patterns do not drastically change day-by-day, and that whether they are asleep or awake at a specific hour can be predicted with good accuracy by time alone. This is an important result, as it shows that the baseline performance, defined by the time-of-day model, is significantly higher than the chance level of 67-71%, calculated by assuming that the average sleep duration across the individuals is between 6 h and 7 h. Therefore, it is necessary that when we report the accuracy of sleep detection algorithms, we compare them to the accuracy of a model only trained on time of the day. This comparison makes the assessment of future sleep detection algorithms easier.

### Limitations

There are a number of limitations that should be considered when interpreting the results of our study. First, the self-reported sleep times are not necessarily accurate themselves. In fact, we observed that a number of participants misreported their sleep start times by a substantial amount; when we fixed these reports, the accuracy of the sleep detection algorithm increased substantially. Apart from directly addressable issues like this, there are many other ways in which self-reports might have been inaccurate. Self-reported sleep start times are in general biased, and people tend to over-estimate their sleep duration [28]. Therefore, what we calculate as accuracy is relative to an inaccurate measure. It may be difficult, both here and in other sleep detection work, to calculate the true accuracies of the algorithms.

Second, the parameters of the HMM were adjusted under the assumption that going to sleep and waking up occur only once in 24 h. Although this assumption is true for most people, there are a number of cases for which it is violated. First, most elderly suffer from fragmented sleep [29], during which they can stay awake for a few hours before going to sleep again. Some sleep pattern disorders, such as insomnia or sleep apnea, cause both segmentation of sleep at night and sleepiness during the day [30], likely affecting daytime behavior as well as sleep patterns. In more extreme cases, such as sleepwalking, patients manifest night-time behaviors that resemble daytime, routine activities [31]. Second, we did not ask whether any of our participants worked in different shifts across days, and some of the anomalies seen in the reported times could have been due to shift-working. Finally, a good number of people, almost one-third of Americans, take day-time naps [32]. Therefore, in many cases, the assumption that a person goes to sleep only once in a 24-h period is incorrect, and further understanding of both population and individual sleep habits will be necessary to create more accurate models.

Third, we do not know if any, or which of these participants had a sleep disorder. People with sleep disorders can be significantly different from the healthy populations in many aspects of their life, which can influence the relationship between mobile phone sensor features and sleep patterns. For example, individuals with disturbed sleep report lower quality of physical functioning, social functioning, vitality, and general health [33]. These differences would likely result in differences in how individuals interact with their mobile phones, thus affecting the data and algorithms for sleep detection. Thus, caution must be applied in generalizing these results to those with atypical sleep patterns.

Finally, our participants were not a perfect sample of the general population in the United States. First, close to 82.6% of our participants were female. Second, we only recruited participants who had WiFi Internet access on their mobile phones. This was important, as the high frequency sensor data can quickly accumulate on the phone and reach the storage limits. Using WiFi to off-load data is energy-efficient and free, unlike using cellular connectivity, which can drain the battery and incur data use fees. For this reason, we recruited participants who had reliable Internet access on their phones. However, with this restriction, it is likely that participants with lower incomes are excluded from our study, who might have different sleep patterns and behavior. Third, 21 participants (10%) changed their phones during the study. Although this may be due to chance, it may also be related to the holiday season, during which people may have received phones as gifts. Finally, we specifically excluded participants with positive screens for several severe psychiatric conditions, which may alter sleep patterns. Thus, it is possible that any or all of these biases reduce the generalizability of these results.

### Comparison With Prior Work

We extended previous research in two important ways. First, our sample size was large relative to previous studies and the study participants were more diverse in age, education level, employment, and location. Although a more diverse sample potentially provides a better training dataset for machine learning, it introduces a few problems. First, diversity means more variability in behavior. Unlike college students who have been the participants of a number of previous studies [11,13], participants from the general population do not necessarily use their mobile phones in ways that can help the sleep detection algorithms. For example, mobile phone usage is one feature that is very useful in detecting sleep states since most people use their phones frequently throughout the day. However, phone usage patterns are different across different age groups. Whereas 22% of Americans aged between 18 and 29 years use their phones every few minutes, this number for an older age group of 50-64 years is only 6% [34]. Therefore, a large and diverse sample introduces new challenges to sleep detection algorithms.

The second way in which we extended the previous research was that we did not give participants any instructions regarding the placement of the mobile phone. This meant that participants, for example, could turn their phones off during sleep, or leave it unplugged so that it runs out of battery. As a result, we found that there were many more missing data points during sleep than during awake states. This, however, was not the only scenario that challenged the sleep detection algorithms. Participants could also leave their phones unattended during the day, or put it in another room when sleeping. Despite all these, the performance of the classifiers is close to, albeit slightly worse than, what has been reported by previous research in more controlled settings.

### Conclusions

As mobile phone technology advances, we expect many of the issues we encountered in this study will vanish. For instance, several of the technical problems we experienced will be ameliorated by longer battery life, standardized hardware, and improved app design. Many other limitations, however, will not be solved by advancing underlying technology. Here we encountered several obstacles, from behaviors that misled algorithms, to sleep patterns unaccounted for by typical models, to inaccurate ground truth data that were due to errors and biases in self-reports rather than technology. Although these obstacles are typically not encountered during demonstrations of sleep detection algorithms, they will likely prove to be impediments to generalized sleep tracking. We believe that mobile phone-based sleep detection technology must tackle these problems in order to become a reliable tool in people’s natural life settings.

## Abbreviations

API: application program interface
AUDIT: alcohol use disorder identification test
BDI: Beck depression inventory
DAST: drug abuse screening test
EEG: electroencephalogram
EMA: ecological momentary assessment
FFT: Fast Fourier Transform
FPG: Focus Pointe Global
GAD: generalized anxiety disorder
HMM: hidden Markov model
MAC: media access control
MAD: median absolute deviation
MDQ: mood disorder questionnaire
PHQ: patient-health questionnaire
USB: Universal Serial Bus
