# Authorship Correction: Scalable Passive Sleep Monitoring Using Mobile Phones: Opportunities and Obstacles

**DOI:** 10.2196/jmir.7932

**Published:** 2017-04-28

**Authors:** Sohrab Saeb, Thaddeus R Cybulski, Stephen M Schueller, Konrad P Kording, David C Mohr

**Affiliations:** ^1^ Center for Behavioral Intervention Technologies Department of Preventive Medicine Northwestern University Chicago, IL United States; ^2^ Rehabilitation Institute of Chicago Department of Physical Medicine and Rehabilitation Northwestern University Chicago, IL United States

The authors of the paper entitled “Scalable Passive Sleep Monitoring Using Mobile Phones: Opportunities and Obstacles” [J Med Internet Res 2017;19(4):e118] inadvertently omitted Stephen M Schueller, PhD (Center for Behavioral Intervention Technologies, Department of Preventive Medicine, Northwestern University) from the list of authors. The author Stephen M Schueller should have been added after Thaddeus R Cybulsky in the published manuscript. Stephen M Schueller was not on the initial authors list at the time of the first submission, and was added during the revising of the article, when he contributed to the writing of the revised manuscript. His name already appears in the authors' funding acknowledgements section in the published paper, “Author SMS was supported by the research grant K08MH102336 from the National Institute of Mental Health.” This omission has been corrected in the online version of the paper on the JMIR website on April 28, 2017, together with the publication of this correction notice. Because this was made after submission to PubMed, the correction notice has been submitted to PubMed. The corrected metadata have also been resubmitted to CrossRef.

## Appendix

Multimedia Appendix 1 Letter from authors requesting correction.

