# Supplementary material for: Authorship Correction: Scalable Passive Sleep Monitoring Using Mobile Phones: Opportunities and Obstacles
Source: J Med Internet Res. 2017 Apr 28;19(4):e143. doi: 10.2196/jmir.7932 (PMC6304214; doi:10.2196/jmir.7932)
Supplement: Multimedia Appendix 1 [file jmir_v19i4e143_app1.pdf]

4/26/17

Dear Editor,

We are contacting you regarding our recently published paper in JMIR, "Scalable Passive Sleep Monitoring Using Mobile Phones: Opportunities and Obstacles". We wanted to ask for the addition of our co-author's name, **Dr. Stephen M Schueller**, whose name was not included in the authors list by mistake during the submission process. The reason for this mistake was that Dr. Schueller was not in the initial authors list during the first submission, and was added during the revision where he contributed to the writing of our revised manuscript. His name is already mentioned in the authors' funding acknowledgements section in the published paper "Author SMS was supported by the research grant K08MH102336 from the National Institute of Mental Health".

We, authors of the above-mentioned paper, agree with the addition of Dr. Schueller's name to our paper. The new order of the authors name will be as following:

Sohrab Saeb, PhD
Thaddeus R Cybulski, BSc
Stephen M Schueller, PhD
Konrad P Kording, PhD
David C Mohr, MD, PhD

And here are the metadata information for Dr. Schueller:

NAME: Stephen M Schueller, PhD
INSTITUTION: Center for Behavioral Intervention Technologies, Department of Preventive Medicine, Northwestern University
ADDRESS: 750 N Lake Shore Dr., Rubloff Building, 10th floor
CITY: Chicago, IL, 60611
COUNTRY: US
ORCID: 0000-0002-1003-0399

We thank the JMIR editor and staff for the time and effort they put in the publication of this paper.

Sincerely yours,

Authors

Sohrab Saeb | Thaddeus R Cybulski | Stephen M Schueller

Digitally signed by Konrad Kording
DN: cn=Konrad Kording, o, ou, email=kk@northwestern.edu, c=US
Date: 2017.04.27 10:43:08 -05'00'

DCMohr (by S Saeb)

Konrad P Kording | David C Mohr
